# Mental health–related structural stigma and discrimination in health and social policies in Nepal: A scoping review and synthesis

**DOI:** 10.1017/S2045796023000823

**Published:** 2023-12-13

**Authors:** D. Gurung, M. Neupane, K. Bhattarai, B. Acharya, N. C. Gautam, K. Gautam, S. Koirala, K. Marahatta, P. Gurung, K. B. Khadka, B. A. Kohrt, G. Thornicroft, P. C. Gronholm

**Affiliations:** 1 Transcultural Psychosocial Organization (TPO) Nepal, Kathmandu, Nepal; 2 Centre for Global Mental Health and Centre for Implementation Science, Institute of Psychiatry, Psychology and Neuroscience, King’s College London, London, UK; 3 Center for Global Mental Health Equity, The George Washington University School of Medicine and Health Sciences, Washington, DC, USA; 4 World Health Organization (WHO) Country office for Nepal, Nepal; 5 National Indigenous Disabled Women Association Nepal (NIDWAN), Nepal; 6 Gandaki Province Health Directorate, Pokhara, Nepal

**Keywords:** discrimination, mental health, Nepal, policy review, structural stigma

## Abstract

**Aims:**

National policies can be used to reveal structural stigma and discrimination in relation to mental health. This review assesses how structural stigma and discrimination are manifested in the policies and legislations of Government of Nepal.

**Methods:**

Scoping review methodology was followed to review policy documents (acts of parliament, legislation, policies, strategies, guidelines and official directives) drafted or amended after 2010.

**Results:**

Eighty-nine policies were identified related to health, social welfare, development and regulations which were relevant to people with psychosocial and mental disabilities or have addressed the mental health agendas. Several critical policy failings and gaps are revealed, such as the use of stigmatizing language (e.g., ‘insane’ or ‘lunatic’), inconsistencies within and between policies, deviation from international protocols defining legal capacity and consent, lack of inclusion of the mental health agenda in larger development policies and lack of cost-effective interventions and identification of financing mechanisms. Provisions for people living with mental health conditions included adequate standard of living; attaining standard mental health; the right to exercise legal capacity, liberty and security; freedom from torture or discrimination; and right to live independently. However, other policies contradicted these rights, such as prohibiting marriage, candidacy for and retention of positions of authority and vulnerability to imprisonment.

**Conclusion:**

Mental health–related structural stigma and discrimination in Nepal can be identified through the use of discriminator language and provisions in the policies. The structural stigma and discrimination may be addressed through revision of the discriminating policies, integrating the mental health agenda into larger national and provincial policies, and streamlining policies to comply with national and international protocols.

## Introduction

For people with lived experience of mental health conditions (PWLE), stigma can result in adverse repercussions in their lives, families and society. Stigma can be defined as problems of knowledge, attitude and behaviours that arise due to perceived biases against PWLE (Thornicroft *et al.*, 2007). Stigma has been highlighted as a key barrier to accessing treatment (Schomerus *et al.*, 2019). It also infringes on the basic human rights of PWLE, thus exacerbating marginalization and exclusion (Thornicroft *et al.*, 2022).

Two decades ago, it was noted that most stigma research focused on frameworks and pathways at the personal level looking into the internalized and interpersonal interactions, while ignoring the macro-level issues underlying stigma (Link and Phelan, 2001). Although much is known about individual experiences of stigma, frameworks that ignore the multi-level mechanisms of stigma limit the understanding of what factors influence and mediate stigma processes (Stangl *et al.*, 2019). Therefore, more studies and actions are needed to consider the macro-level factors and mechanisms of stigma – also called ‘structural stigma’ (Hatzenbuehler and Link, 2014; Thornicroft *et al.*, 2022).

Structural stigma is the ‘societal-level conditions, cultural norms, and institutional practices that constrain the opportunities, resources, and wellbeing for stigmatized populations’ (Hatzenbuehler and Link, 2014). It refers to policies and practices causing intentional or unintentional disadvantages for the stigmatized population, thus creating and perpetuating social inequities for PWLE (Knaak *et al.*, 2020). Empirical research on structural forms of stigma and discrimination comes from social and political sciences that investigates its negative outcomes on sexual and racial minorities. However, there is a dearth in understanding structural stigma in relation to mental health conditions (Hatzenbuehler *et al.*, 2010). Some studies have tried to address this gap by documenting structural discrimination in legal provisions such as prohibition in marriage and voting, violation of property rights and involuntary admissions that have violated the rights of persons with psychosocial and mental disabilities (Bhugra *et al.*, 2016; Conley and Baum, 2023).

Since the 1990s, Nepal has seen changes in the mental health policy landscape. Amidst political and environmental disasters, the mental health sector has undergone reforms (Singh and Khadka, 2022). The first national mental health policy was formed in 1996; however, its status of implementation was questionable. The Bhutanese refugee situation and the Maoist conflict highlighted the need for psychosocial and mental healthcare in Nepal. After the earthquake in 2015 and after the prioritization of mental health in United Nation’s Sustainable Development Goals, the mental health agenda gained momentum in Nepal with its inclusion in the nation’s development plan and designation of a division in the Department of Health Services as a focal unit to oversee mental health programmes. This led to the drafting of a national community mental healthcare package that was brought into implementation in 2017 (Government of Nepal, 2017b).

After the inclusion of mental health into the National Health Policy in 2019, a National Mental Health Strategy and Action Plan was developed in 2020, which remains the only policy document that solely addresses mental health (Government of Nepal, 2021a). Despite these reforms, the mental health sector faces numerous challenges. At least 9 of 10 people with mental health conditions in Nepal do not access mental health treatment, and stigma has been identified as the leading barrier in access to care (Luitel *et al.*, 2019, 2017). Stigma is reflected in the structures of Nepali society such as the policy environment and these structures, along with cultural norms of ‘what matters most’ such as productivity, prestige and acceptance, further driving stigma in Nepal (Gurung *et al.*, 2022). Hence, the aim of this review is to understand the provisions for PWLE in Nepal’s policies and legislations and assess how structural stigma and discrimination are manifested within these policies.

## Methods

We conducted a scoping review using the Arksey and O’Malley scoping review method that suggests defining the question, identifying, selecting mapping data, summarizing, synthesizing and reporting findings (Arksey and O’Malley, 2005). We reviewed National and Provincial policies using the following guiding questions:

1. How are mental health issues and persons with lived experience of mental health conditions and psychosocial disabilities (PWLEs) labelled and defined in these policies?
2. What are the provisions that promote the rights of PWLEs?
3. What are the notable gaps in the policies that restrict the basic human rights of PWLEs?

### Search and screening strategy

We conducted systematic searches in three government websites: (i) Ministry of Health and Population (MoHP) (https://mohp.gov.np/en); (ii) Ministry of Women, Children, and Senior Citizens (MoWCSC) (https://mowcsc.gov.np/en); and (iii) Nepal Law Commission (https://lawcommission.gov.np/en/). Additionally, DG emailed 10 stakeholders and policymakers working in the field of mental health to identify and share relevant policy documents.

Each document available in English was screened in two steps. First, documents were screened for their mention of terms related to mental health, discrimination, psychosocial/psychological and disability (using the terms ‘mental’, ‘discrim*’, ‘psych*’and ‘disab*’). Second, they were screened to assess if they met the inclusion criteria (see text box 1 for the inclusion criteria). The documents available only in Nepali were assessed by reviewers fluent in Nepali to screen if they met the inclusion criteria. Full review was conducted for Nepali-only documents by reviewers as using search terms in Nepali fonts was not feasible. Document search and screening were carried out from February to December 2021; documents collected via emails from stakeholders were screened until April 2022 (Table 1).

**Table 1.** Inclusion criteria for screening policy documents

Laws, legislations, acts, policies, guidelines, directives, rules, regulations, programmes, strategies, plans, orders/bills drafted or passed by institutions and bodies under the Government of Nepal which contain any text related to mental or psychosocial or psychological health/disability/services/programmes are available in English and/or Nepali were drafted or amended after 01 January 2010 to 31 April 2022 [The year after 2010 was chosen (for drafting and amendment) because due to the then political scenario of Nepal (such as drafting of interim constitutions and transition to federal system of government) most of the relevant policies that were being implemented at the time had gone through amendment].

### Extraction and synthesis

Three reviewers (DG, NCG and KB) extracted information from the documents that met inclusion criteria into a data extraction sheet that recorded their type, year of information, year of final amendment, inclusion of key terms and texts related to the key terms within the document. As our definition of the policy included a range of documents from laws to directives issued by the government and covered not just mental health but also general health, social welfare and regulatory policies, following just one policy analysis framework was not feasible. Therefore, we developed a separate framework adapted from indicators recommended by WHO in the mental health legislation, policy and plan checklists and in the WHO QualityRights toolkit (World Health Organization, 2012).

The adapted framework consisted of the following themes that were used to code and synthesize the extracted text: (i) language and definitions; (ii) expansion and contraction of rights and liberties; (iii) integration and consistency with the national policy and legislative environment; (iv) coordination and inter-sectoral collaborations; and (v) financing. Most of the policy documents were reviewed in their English-translated format made available by the Law Commission website. However, for the language and definitions, both the official Nepali version of the documents along with their English-translated documents were reviewed to identify stigma terminology. Any questions and/or disparities were discussed among the reviewers to form a consensus and reduce subjectivity. After data extraction and mapping, DG carried out a synthesis of the results and shared with the review team for inputs.

## Results

### Policy selection

**Figure 1.**
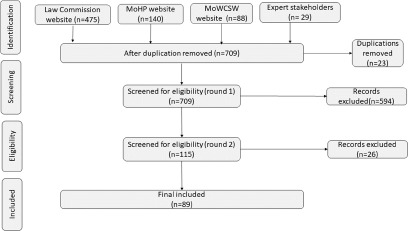
PRISMA search strategy for scoping reviews. PRISMA: Preferred Reporting Items for Systematic Reviews and Meta-Analyses.

There were 475 policies listed in the Law Commission website, 140 in the MoHP and 88 in the MoWCSC website (Fig. 1). In addition, 29 potentially relevant documents were sourced via key stakeholders. After removing 23 duplicates, 709 policy documents were included for screening. We removed 594 policy documents after initial screening because they had not been drafted or updated since 2010 or because those documents did not include any terms related to mental or psychosocial or psychological health/disability/services/programmes. Documents were removed when a contemporary policy overriding the older one was identified. The remaining 115 policy documents were reviewed in full text, after which 26 were removed for not having provisions that were directly related to psychosocial and mental health/disabilities or affected the people with psychosocial and mental health conditions. A total of 89 policy documents were included in the study for data extraction and synthesis (Table 2).

**Table 2.** Policy documents included in the review

Document Number	Name of policy, act, law with Nepali year (Gregorian year)	Source	Date drafted in Gregorian calendar	Date amended in Gregorian calendar	Type of document	Languages available	Brief information on documents
1	Army Act, 2063 (2006) Amended (2010)	Law Commission website	2006	2010	ACT	English/Nepali	This act is relating to the establishment, arrangement, control, use and mobilization of the Nepal Army for making the Nepal Army accountable to the people of Nepal.
2	The Act relating to Children, 2075 (2018)	Law Commission website	2018	N/A	ACT	English/Nepali	Act formulated to maintain the best interests of the children, by respecting, protecting, promoting and fulfilling the rights of the child.
3	Citizen Investment Trust Act, 2047 (1991)	Law Commission website	1991	2010	ACT	English/Nepali	This act is under existence to increase opportunities of investment upon encouraging including general public for capital saving and to bring dynamism in the development of capital market.
4	Nepal Citizenship Act 2063 (2006)	Law Commission website	2006	2017	ACT	English/Nepali	This act is all about procedures for obtaining certificate of citizenship of Nepal.
5	Civil service Act, 2049 (1993)	Law Commission website	1993	2010	ACT	English/Nepali	This act is made to make the civil service more competent, vigorous, service-oriented and responsible.
6	Consumer protection act, 2075 (2018)	Law Commission website	2018	N/A	ACT	English/Nepali	This act is made for the purpose of protecting the rights of consumers, managing the market in accordance with the market rule and making market fair and transparent, the levels of market involved in taking the prescribed goods or services from the producer or importer to the final consumer
7	Cooperatives Act, 2017	Law Commission website	2017	N/A	ACT	English/Nepali	The act was made to make provisions on the formation and operation of various types of cooperative associations and societies based on the mutual support and cooperativeness for the economic and social development of the public at large.
8	Domestic Violence (Offence and Punishment) Act, 2066 (2009)	Law Commission website	2009	N/A	ACT	English/Nepali	This act is made to respect the right of every person to live in a secure and dignified life, to prevent and control violence occurring within the family and for matters connected there with and incidental there to making such violence punishable, and for providing protection to the victims of violence.
9	Education Act, 2028 (1971)	Law Commission website	1971	2010	ACT	English/Nepali	This act is made for the improvements in the management of existing and future schools all over Nepal in order to prepare human resource for national development and to maintain good conduct, decency and morality of the people in general in consonance with multi-party democratic system.
10	Impeachment (Regulation of Procedure) Act, 2059 (2002)	Law Commission website	2002	2010	ACT	English/Nepali	This act is made to take action against corruption by filing against him/her pursuant to prevailing law.
11	Local Level Election Act 2017	Email from the stakeholders	1992	2010	ACT	English/Nepali	This act is under prevalent to ensure that voter, whose name is registered in the Electoral Rolls of any ward of a Rural Municipality or Municipality, shall only be entitled to vote in the polling.
12	National Human Rights Commission Act, 2068 (2012)	Law Commission website	2012	N/A	ACT	English/Nepali	This act is made to ensure the respect, protection and promotion as well as effective implementation of human rights
13	Nepal Airlines Corporation Act, 2019 (1963)	Law Commission website	1963	2010	ACT	English/Nepali	This act is made for the following provision. A Corporation shall be established in the name of Nepal Airlines Corporation. (2) The Corporation shall be an autonomous corporate body having perpetual succession. This corporation shall have its own separate seal for all of its business
14	Nepal Health Service Act, 2053 (1997)	Law Commission website	1997	2010	ACT	English/Nepali	This act is made to make the health service more competent, vigorous, service-oriented and responsible
15	Administration of Justice Act, 2073 (2016)	Law Commission website	2016	N/A	ACT	English/Nepali	This act is enacted for amendment and unification of the law relating to administration of justice
16	Judicial Council Act, 2073 (2016)	Law Commission website	2016	N/A	ACT	English/Nepali	This act is enacted to Amend and Consolidate Legislation Relating to Function,Duty and Power of Judicial Council
17	Nepali Language Publication Corporation Act, 2021 (1964)	Law Commission website	1964	2010	ACT	English/Nepali	This act is made to establish and provide for the Nepali Language Publication Corporation and made it as a high publication body by publishing such books, etc.
18	Police Act, 2012 (1955)	Law Commission website	1955	2010	ACT	English/Nepali	This act is under provision for the Formation and Arrangement of Nepal Police Force and Specification of the Functions and Duties of Police Personnel
19	Prisons Act, 2019 (1963)	Law Commission website	1963	2010	Act	English/Nepali	This act is made to amend and codify Nepal laws pertaining to prison in order to maintain law and order
20	The Act Relating to Rights of Persons with Disabilities, 2074 (2017)	Law Commission website	2017	2018	ACT	English	This act is made to amend and consolidate laws relating to rights of persons with disabilities in order to respect their civil, political, economic, social and cultural rights by doing away with discrimination against persons with disabilities and to ensure the environment that enables persons with disabilities to earn self-reliant and respectful living by empowering persons with disabilities and getting them to have participation in the process of policy making, and development
21	Public debt Act 2059 (2002)	Law Commission website	2002	2010	ACT	English/Nepali	This act came into existence for the Raising of Public Debts by Issuing Bonds
22	Remuneration and Facilities of Authorities and Members of Parliament Act, 2052 (1996)	Law Commission website	1996	2010	ACT	English/Nepali	This act came into existence to assist the authorities and member of parliament on the facilities and remuneration
23	Public Health Service Act, 2075 (2018)	Law Commission website	2018	N/A	ACT	English/Nepali	This act is made to make necessary legal provisions for implementing the right to get free basic health service and emergency health service guaranteed
24	The right to employment act, 2075 (2018)	Law Commission website	2018	N/A	ACT	English/Nepali	This act is formed to provision rights of citizens to voluntarily engage in employment according to the qualification and capacity and leave or change such employment,
25	The Right to Safe Motherhood and Reproductive Health Act, 2075 (2018)	Law Commission website	2018	N/A	ACT	English/Nepali	This act explains that no one shall discriminate on the right to get monthly services including family planning, reproductive health, safe motherhood, safe abortion, emergency obstetric and newborn care, morbidity on the ground of one’s origin, religion, color, caste, ethnicity, sex, community, occupation, business,
26	The Social Security Act, 2075 (2018)	Law Commission website	2018	N/A	ACT	English/Nepali	This act is under existence for the right to get the social security allowance: (a) Senior citizens, (b) Indigent, (c) Incapacitated and helpless persons, (d) Helpless single women, (e) Citizens with disabilities, (f) Children, (g) Citizens unable to take care themselves.
27	The Right to Housing Act, 2075 (2018)	Law Commission website	2018	N/A	ACT	English/Nepali	This act is under existence to provide housing facility from domestic private sector: (1) The private company or body corporate may construct housing and distribute it to the citizen, subject to the prevailing law. (2) Minimum service, facility and infrastructure shall have to be available in the housing referred to in sub-section (1).
28	The Act Relating to Compulsory and Free Education, 2075 (2018)	Law Commission website	2018	N/A	ACT	English/Nepali	This act came into existence to provide compulsory and free education for all Nepalese. Includes compulsory education for children from the ages of 5 to 13 years. Implementing text(s):
29	Crime Victim Protection Act, 2075 (2018)	Law Commission website	2018	N/A	ACT	Nepali only	This act is under existence to protect the rights of victim. Victim shall have the right to enjoy decent, fair, dignified and respectful treatment during the criminal justice process. status, age, physical or mental unsoundness, disability or ideology or similar other ground.
30	The Privacy Act, 2075 (2018)	Law Commission website	2018	N/A	ACT	English/Nepali	This act is made as the colour first specific legislation of Nepal governing the protection of individual privacy.
31	Sports Development Act, 2048 (1992)	Law Commission website	1992	2010	ACT	English/Nepali	This act is under existence to prepare competent and disciplined citizens by having physical and mental development of students and general public through sports and to develop sports by making necessary provisions, appropriate participation of Nepal in sports competitions to be held at the international level;
32	Drugs Control Strategy, 2010	Law Commission website	2010	N/A	Strategy	English/Nepali	This strategy is to help attain the objectives set out by the National Policy for Drugs Control, 2006.
33	National Youth Policy, 2010	Law Commission website	2010	N/A	Policy	English/Nepali	This policy is to rendering necessary support to the vulnerable youths, bringing out the capacity inherent in them, making appropriate management to prevent brain drain of youths and developing further potentiality, while ensuring the access of the Nepalese youths to opportunities generated globally
34	Domestic Violence (Offence and Punishment) Rules, 2067 (2010)	Law Commission website	2010	N/A	Rules	English/Nepali	The rules respect the right of every person to live in a secure and dignified life, to prevent and control violence occurring within the family and for matters connected there with and incidental there to making such violence punishable, and for providing protection to the victims of violence;
35	Motor Vehicles and Transport Management Rules, 2054 (1997) and act (1993)	Law Commission website	1997	2010	Rules	English/Nepali	This rule is made to make transportation services consolidated, efficient and effective with a view to preventing motor vehicle accidents, enabling the victims of accidents to have compensation, providing for insurance and making transportation facilities available to the public generally in a simple and easily accessible manner;
36	Public Debt Rules, 2059 (2003)	Law Commission website	2003	2011	Rules	English/Nepali	Public debt rules are formed to ensure that every citizen pay their debt on time
37	The Constitution of Nepal	Email from the stakeholders	2015	NA	Rules	English/Nepali	Fundamental Law of Nepal. The constitution of Nepal is divided into 35 parts, 308 Articles and 9 Schedules. This constitution is here to fulfil the aspirations for perpetual peace, good governance, development and prosperity through the medium of federal democratic republican system of governance, hereby promulgate this Constitution through the constituent Assembly
38	National Health Policy 2076	Ministry of Health and Population Website	2019		Policy	English/Nepali	Formulated to protect the achievement made so far and to adequately address prevalent and new challenges faced within health sector by developing people’s centred and efficient management by optimum utilization of available means and resources to provide promotive, preventive, curative and rehabilitative health services.
40	Criteria for appointment and nomination of officials and members of public bodies affiliated to the Ministry of Health 2073	Ministry of Health and Population Website	2017	N/A	Criteria	Nepali only	This Directive is made to prioritise the appointment and nomination of government officials and member of public bodies on the basis of fair, competition, inclusion and many other criteria to be fulfilled.
41	National Strategy for Reaching the Unreached	Ministry of Health and Population Website	2016	N/A	Strategy	Nepali only	Strategy for reaching the unreached is here with an objective to reduce health and nutrition inequalities and contributing toward Universal Health Coverage in Nepal
42	National Consolidated Guideline for Treating and Preventing HIV in Nepal	Ministry of Health and Population website	2014	N/A	Guideline	English/Nepali	It is made with an objective to provide updated, evidence-based clinical recommendations outlining a public health approach to providing antiretroviral (ARV) drugs for HIV treatment and prevention in the context of the continuum of HIV care, Ministry of Health and Population, National Center for AIDS & STD Control revised the National ART Guidelines developed in 2012, based on the recommendations adopted to Nepal context from WHO ‘Consolidated Guideline on The use of ARV Drugs for Treating and Preventing HIV Infection’
43	National Guidelines on Case Management of STI Final December 2014	Ministry of Health and Population website	2014	N/A	Guideline	English/Nepali	This guideline is made realizing the importance of controlling STIs as a major public health intervention an in view of making the syndromic approach a standard practice in the health institutions of the country
44	National HIV testing and Treatment Guidelines 2017	Ministry of Health and Population website	2017	N/A	Guideline	English/Nepali	This guideline is made to conduct safe and effective HIV testing services, innovative service delivery approaches in ‘Nepal HlVision 2020’ include intensified testing to reach key populations through facility-based outreach and community-led in-reach; linking testing to treatment.
45	National Training Manual on the Management of Sexually Transmitted Infections	Ministry of Health and Population website	2011	N/A	Guideline	English/Nepali	National Training Manual on the Management of Sexually Transmitted Infection is made to facilitate the training of medical personnel (medical doctors,staff nurses, health assistants and other paramedics) at various levels to deliver adequate and quality sexually transmitted infection (STI) services in areas with limited resources as well. T
46	National Operating Guide for Leprosy Program	Ministry of Health and Population website	2018	N/A	Guideline	Nepali only	Operating guide for leprosy program is made with an aim of early detection of new cases and timely complete treatment with multi drug therapy (MDT) through integrated health services. Major target for 2020 is to reduce grade II disability below 1 per million populations.
47	National guidelines and standard operating procedures for home and community-based care and support programmes in HIV and AIDS	Ministry of Health and Population website	2011	N/A	Guideline	Nepali only	Guidelines and standard operating procedures for home and community-based care and support programmes is made with an aim to better support to effectively serve those infected and affected by HIV in Nepal
48	SSU, Geriatric Ward, Gender Equity and Social Inclusion (GESI)-related program operation guidance	Ministry of Health and Population website	2018	N/A	Guideline	Nepali only	Operation guidelines on gender equality and social inclusion are made as a new opportunity for sectoral ministries to address gender and exclusion and to integrate GESI into their systems and services.
49	Program operation guideline 2076−2077 – province level	Ministry of Health and Population website	2019	N/A	Guideline	Nepali only	Provincial-level program operation guidline is made for the annual programme implementation at province level.
50	Program operation guideline 20762-077 – local level	Ministry of Health and Population website	2019	N/A	Guideline	Nepali only	Local-level program operation guideline is made for the annual programme implementation at local level
51	Disability-related 10 years national policy and action plan 2016−2025	Ministry of Women Children, and Senior Citizens website	2016	N/A	Policy	Nepali only	Disability-related national policy and action plan is drafted with an aim to harmonize the disabled-friendly programmes to be conducted by the public and private sectors, cooperatives, non-governmental organizations and community organizations
52	Allegations of Witchcraft (Offenses and Punishment) Act, 2072	Ministry of Women, Children, and Senior Citizens website	2015	N/A	ACT	Nepali only	Allegations of Witchcraft Act 2072 aims to provide legal recourse for women who have experienced physical or mental harm as a consequence of witchcraft accusations and related cultural and superstitious practices by proposing jail terms of up to seven years and fines up to Nepali Rupees
53	Prevention of Sexual Harassment in the Workplace Act, 2071	Ministry of Women, Children, and Senior Citizens website	2014	N/A	ACT	Nepali only	Prevention of Sexual Harassment in the Workplace is made to make the necessary provisions for the prevention of sexualharassment at workplace by ensuring the right of every person to work in a safe, fair and dignified environment.
54	Gender-Based Violence Prevention Fund Operation Rules, 2067	Ministry of Women, Children, and Senior Citizens website	2010	N/A	Rules	Nepali only	Operation rules for Gender-Based Violence Prevention Fund Operatation operate at all three levels of government under the authority of each level’s laws and through the management of each level’s resources to establish the fund and provide resources and oversight for these funds
55	Guidelines for grants provided to institutions providing services in the women, children and elderly population sector, 2077	Ministry of Women, Children, and Senior Citizens website	2020	N/A	Guidelines	Nepali only	Written guide to assist the institution providing services for women, children and elderly population
56	Rehabilitation Center Operation Guideline First Amendment 2072	Ministry of Women, Children, and Senior Citizens website	2015	N/A	Guideline	Nepali only	To facilitate the rehabilitation and reintegration of the vulnerable persons at the centres
57	Community-Based Rehabilitation (CBR) programme operations directory	Ministry of Women, Children, and Senior Citizens website	2011	N/A	Directive	Nepali only	CBR operations directory is made to ensure the effective implementation of CBR
58	Psychosocial counselling guide for stakeholders involved in the campaign against human trafficking	Ministry of Women, Children and, Senior Citizens website	2011	N/A	Guidelines	Nepali only	Psychosocial counselling guide for stakeholders involved in the campaign against human trafficking is a guideline that facilitate the stakeholder who are involved in the campaign against human trafficking.
59	Child Protection Financial Relief Assistance Procedure, 2076	Ministry of Women, Children, and Senior Citizens website	2016	N/A	Procedure	Nepali only	This procedure aims to address identified needs to safeguard and promote a child’s welfare where there is no other legitimate source of financial assistance.
60	Child Helpline No. 1098 Nepal, Operation Procedure, 2076	Ministry of Women, Children, and Senior Citizens website	2016	N/A	Procedure	Nepali only	Child Helpline number operation procedure help facilitate is the operation of helpline number operated by the Child Workers Concerned Centre in Nepal (CWIN)
61	Child Search No. 104 Operation Guide, 2076	Ministry of Women, Children, and Senior Citizens website	2016	N/A	Guidelines	Nepali only	The National Child Rights Council and Nepal Police jointly operate National Centre for Children at Risk (hotline number 104) for the effective rescue and protection of child
62	Integrated activities related to rescue, protection and management of street children,2076	Ministry of Women, Children, and Senior Citizens website	2016	N/A	Guidelines	Nepali only	This is a guideline to rescue, protect and manage street children for their protection. Psycho-social counselling, socialization, family reunion, rehabilitation and reintegration are integrated to ensure that they do not return to the streets again
63	Procedure for providing grants to children’s homes established for the protection of children with disabilities, 2077	Ministry of Women, Children, and Senior Citizens website	2020	N/A	Procedure	Nepali only	This procedure guide and facilitate in providing grants to children’s homes established for the protection of children with disabilities,
64	‘Standards for Operation and Management of Residential Child Care Homes, 2012’	Ministry of Women, Children, and Senior Citizens website	2012	N/A	Criteria	Nepali only	Standard for Operation and management of residential child care home provide guidelines to run residential Child Care Homes. This has contributed in increasing the number of children living in the residential child care Homes.
65	Criteria for the appointment of members of the Central Senior Citizens Committee, 2075	Ministry of Women, Children, and Senior Citizens website	2018	N/A	Criteria	Nepali only	Criteria for the appointment of members of the central senior citizens committee is made to tackle issues and address the needs of the senior citizen.
66	Criteria regarding the expenditure of conditional grant amount for the establishment and expansion of Gender-Based Violence Prevention Fund in the state, 2076 BS	Ministry of Women, Children and Senior Citizens website	2019	N/A	Criteria	Nepali only	This criterion came into existence to ensure the grant amount is mobilized with a systematic process for the established and expansion of Gender-Based Violence Prevention.
67	Criteria for Expenditure of Conditional Grants for Establishment and Expansion of Gender-Based Violence Prevention Fund at All Local Levels, 2076	Ministry of Women, Children, and Senior Citizens website	2016	N/A	Criteria	Nepali only	The Ministry of Women, Children, and Senior Citizens has developed a standard for expenditure of conditional grants provided to all local levels for the establishment and expansion of Gender Based Violence Prevention Fund.
68	Criteria for Operation of Shelter during Coronavirus (COVID-19) Epidemic, 2077	Ministry of Women, Children and, Senior Citizens website	2021	N/A	Criteria	Nepali only	COVID-19 pandemic posed serious challenges where government struggled in pandemic response plans and to steer the health system ensuring its continued functioning. Therefore, this criterion is prepared to facilitate the operation of shelter during pandemic.
69	National Adolescent health development Strategy	Ministry of Health, Department of Health Service website	2018	N/A	Strategy	English/Nepali	The overall aim of the National Adolescent Health and Development Strategy is to improve the health and socio-economic status of adolescents.
70	Health Research Areas of Nepal 2019	Email from stakeholders	2019	N/A	Others	English/Nepali	Health Research areas of Nepal 2019 is established to promote quality and ethical standard of health research in the country through rigorous and continuous process of review of different national documents, reports, health sector.
71	National Civil Code Act, 2017 (2074)	Email from the stakeholders	2017	N/A	Act	English/Nepali	National Civil Code that includes criminal and civil code. This code makes material changes to the laws of contract and execution formalities of contracts, deeds and other documents to bring major reforms in the country’s legal system.
72	The National Penal (Code) Act, 2017(2074)	Email from the stakeholders	2018	N/A	ACT	English/Nepali	This act is under practice to provide for a timely code on criminal offences, by amending and consolidating the laws in force relating to criminal offences, in order to uphold morality, decency, etiquette,convenience, economic interest of the public, by maintaining law and order in the country, maintain harmonious relationship and peace among various religious and cultural communities, and prevent and control criminal offences
73	NPC 15th Plan 2019/20−23/24	Email from the stakeholders	2019	N/A	Plan	English/Nepali	14th Plan set by the National Planning Commission to meet the national development goals
74	14th Plan of national planning commission (2016/17−18/19)	Email from the stakeholders	2016	N/A	Plan	Nepali only	14th Plans set by the National Planning Commission to meet the national development goals
75	13th plan of NPC 2013/14−15/16)	Email from the stakeholders	2014	N/A	Plan	Nepali only	13th plan set by the National Planning Commission to meet the national development goals
76	12th plan of NPC (2011/12−13/14)	Email from the stakeholders	2011	N/A	Plan	Nepali only	12th plan set by the National Planning Commission to meet the national development goals
77	Multisectoral action plan on the prevention and control of NCD in Nepal 2021−2025	Email from the stakeholders	2021	N/A	Plan	English	The goal of the multisectoral action plan is to reduce preventable morbidity, avoidable disability and premature mortality due to NCDs in Nepal.
78	Operation guideline for health programmes in province level 2075/76	Email from the stakeholders	2018	N/A	Guidelines	Nepali	Ministry of Health and Population published a Program Implementation Guideline for Province 2075/76 to implement various health programmes at province level
79	NHSSP 2 (2010−2015)	Ministry of Health and Population website	2010	N/A	Plan	English	Programmes and strategies Plans set jointly by the Government and development partners for 2010−2015 that aims to address social determinants of health towards achievement of universal health coverage in line with the country’s goals.
80	NHSSP 3 2015−2020	Email from the stakeholders	2015	N/A	Strategy	English	Programmes and strategies Plans set jointly by the Government and development partners for 2015−2020 that aims to address social determinants of health towards achievement of universal health coverage in line with the country’s goals.
81	National Health Service Program implementation plan 2016−2021	Ministry of Health and Population website	2016	N/A	Plan	English	Plans set jointly by the Government and development partners for 2016−2021 that aims to address social determinants of health towards achievement of universal health coverage in line with the country’s goals.
82	Provincial health policy Gandaki Province 2078	Email from the stakeholders	2021	N/A	Policy	Nepali only	Provincial Health Policy for Gandaki Province is made to ensure that all people from Gandaki province, regardless of ethnic, gender, caste, class will be guided by the policy
83	Provincial Health Policy Karnali 2076	Email from the stakeholders	2019	N/A	Policy	Nepali only	Provincial Health Policy for Karnali Province is made to ensure that all people from Karnali Province, regardless of ethnic, gender, caste, class will be guided by the policy
84	National Health Insurance Act	Email from the stakeholders	2017	2019	ACT	Nepali only	Act formed to provide health insurance coverage and insure access to cost effective and quality healthcare for Nepali citizen
85	National Mental Health Strategy and Action Plan	Email from the stakeholders	2020	N/A	Plan	Nepali only	This document provides a blueprint for governments, academics, health professionals, civil society and others for the planning and implementation of mental health programmes.
86	Community mental health package, 2074	Email from the stakeholders	2017	N/A	Package	English	Community mental health package is made to facilitate implementation of community mental healthcare programmes that include integration of mental health into primary healthcare packages
87	Operation guideline for province health programmes 2078/79	Email from the stakeholders	2021	N/A	Guidelines	Nepali only	This is a health programme implementation guideline for the implementation of health programmes by province government
88	Program operation guideline – local level 2078/79	Email from the stakeholders	2021	N/A	Guidelines	Nepali only	This is a health programme implementation guideline for the implementation of health programmes by local government for fiscal year 2078/79 by
89	Standard treatment protocol (STP) for basic health services (BHS) package 2078	Email from the stakeholders	2021	N/A	Protocol	English	Standard treatment protocol (STP) for basic health services (BHS) packages. This STP provides treatment guidance on several diseases included in basic healthcare packages including mental health.

### Language and definitions

We identified 23 of 89 policy documents with stigmatizing terms. The original Nepali terms and the English translations made available in the Law Commission website are shown in Table 3. Since 2015, policies were more likely to use neutral terminology such as ‘persons with psychosocial and mental disabilities’.

**Table 3.** Categories of stigmatizing language and policies endorsing them

Groupings	Terminology in English-translated documents (made available in the Law Commission website)	Terminology in Nepali in an official policy document	Policy documents endorsing one or more instances of stigmatizing terms
Labelling terminology	drug abuser insane mad lunatics person who is mentally unsound mentally sick mentally abnormal	लागु औषधि दुर्ब्यसनि बौलाहा/ बौलाएको पागल र अर्धपगाल मगज बिग्रेको मानसिक सन्तुलन रहित व्यक्ति मानसिक सन्तुलन ठिक नभएको विक्षिप्त अभियुक्त होश ठेगान मा नरहेको	Drugs Control Strategy, 2010 Local Level election act 2017 Army Act, 2006 (amended 2010) Citizen Investment Trust Act, 1991 (amended 2010) Police Act, 1955 (amended 2010) Nepali Language Publication Corporation Act, 1964 (amended 2010) Prisons Act, 1963 (amended 2010) National Human Rights Commission Act, 2012 Judicial Council Act, 2016 Public Debt Act, 2002 (amended 2010) Criteria for the appointment of members of the Central Senior Citizens Committee, 2018 The National Penal (Code) Act, 2017
Vague or imprecise language	mental unsoundness mentally sick drug abuse mentally ill	मानसिक अस्वस्थता मानसिक बिरामी लागु औषधि दुरूपयेfग	Crime Victim Protection Act, 2018 Prisons Act, 1963 (amended 2010) Drugs Control Strategy, 2010
Mental health conditions as afflictions	suffering from a mental illness/mental unsoundness mental disease	मानसिक रोग मानसिक अवस्था ठिक नभएको मानसिक अवस्था बेहोरिरहेका]	The Social Security Act, 2018 National Youth Policy, 2010 National Operating Guide for Leprosy Program, 2018
Terminology implying incompetence	mentally incapable mentally incompetent mentally incapacitated	मानसिक रुपमा काम गर्न नसक्ने मानसिक रुपले असक्त	Cooperatives Act, 2017 Remuneration and Facilities of Authorities and Members of Parliament Act, 1996 (amended 2010) The Social Security Act, 2018
Dated terminology	mentally handicapped mentally disabled mental retardation	मानसिक रुपले असक्त सुस्त मनस्थिति	National Civil Code, 2017 The Social Security Act, 2018

We found no policy documents that formally provided definitions of mental health conditions, mental disorders or psychosocial problems. This could be because there is no legislation specific to mental health. The commonly used terminology that relates to persons with mental health conditions included in most of the social and civil policy documents is ‘of unsound mind’ (Nepali: *hosh thegan ma naraheko*). This is a legal terminology that is defined in the National Penal (Code) Act, 2017 as ‘… the condition of being incapable due to physical and mental ill health, of knowing the act done by oneself in general understanding and consequences thereof’ (Government of Nepal, 2017e). Other stigmatizing terms such as ‘insane’, ‘mentally unsound person’ and ‘lunatic’ were used in some of the civil policies.

Most policies mentioned health in general without distinguishing between mental health and physical health. The Public Health Service Act, 2017 (Government of Nepal, 2018f) mentions health services related to ayurveda, allopathy, homeopathy and other natural treatments but does not mention psychosocial therapies and counselling services. Policy documents have not distinguished different types of mental disorders, and rather use overarching terms such as mental health/mental disorders, and mental disability.

Some policy documents included psychosocial and mental disabilities in their definitions of disability. The closest legislative document that relates to mental health is the Act Relating to the Rights of Persons with Disabilities (will be referred to as the Disability Act henceforth) (Government of Nepal, 2017a). The Disability Act has further defined disability as – ‘a person who has long- term physical, mental, intellectual, or sensory disability, or functional impairments, or existing barriers that may hinder his or her full and effective participation in the social life on an equal basis with others’. However, the definition and difference between ‘persons with psychosocial’ and ‘persons with mental’ disabilities are missing here too. The Disability Act in the classification of disabilities has further defined mental or psycho-social disability as ‘the inability to behave in accordance with age and situation and delay in intellectual learning due to problems in performing intellectual activities like problems arising in the brain and mental parts and awareness, orientation, alertness, memory, language, and calculation’. This definition, however, focuses on developmental and intellectual disability but fails to capture the range of other psychosocial and mental disabilities.

### Expansion or contraction of rights and liberties

The expansion or contraction of rights and liberties of PWLEs in the policy documents is summarized in five themes based on the WHO QualityRights Tool Kit: (i) right to an adequate standard of living; (ii) right to the enjoyment of highest attainable standard of physical and mental health; (iii) right to exercise legal capacity and right to personal liberty and security; (iv) freedom from torture or cruel, inhuman, or degrading treatment or punishment and from exploitation, violence, discrimination and abuse; and (v) right to live independently and be included in the community.

#### Right to adequate standard of living

This sub-theme includes the right to privacy, housing and social security for PWLEs addressed in various policy documents.

The Right to Housing Act does not directly address protection of housing for PWLEs. However, it notes that ‘No citizen shall be made deprived of, or discriminated from, the facility of housing on the ground of origin, religion, class, caste, ethnicity, gender, physical condition, disability, health condition, marital status, pregnancy, economic condition, language or region, ideology or any other such grounds’ (Government of Nepal, 2018h). It has special consideration for eviction due to public purposes for persons with disability, helpless and elderly citizens, although provisions for homelessness due to mental health conditions are not discussed. The Disability Act does mention housing programmes for persons with psychosocial disabilities who have been disregarded by their family members (Government of Nepal, 2017a). It does not outline who, where and how they will be housed or rehabilitated.

The Social Security Act has adopted the definition of disability to include mental and intellectual disability and has defined ‘incapacitated and helpless’ as those who are incapacitated to become economically productive due to physical or mental conditions and have no family members capable of taking care of them (Government of Nepal, 2018i). The act mentions that these citizens certified by doctors as suffering from (amongst others) ‘*mental retardation*’ and ‘*mental unsoundness*’ are entitled to social security which includes allowances specified by the government. The Privacy Act has provision for privacy of information regarding physical and mental health conditions of each person as inviolable except in matters of consent of the concerned person, in which case such information can be disclosed (Government of Nepal, 2018e).

#### Right to enjoyment of highest attainable standard of physical and mental health

This sub-theme encapsulates provisions on the availability of treatment, skilled staff, service user–driven treatment and rehabilitation, availability of medication, adequate services available for general and reproductive health, and various interventions highlighted in the policy documents.

The constitution of Nepal states that all citizens have the right to free basic health service from the state (Government of Nepal, 2015), and the Public Health Service Act includes services relating to ‘mental disease’ (Government of Nepal, 2018f). Most policy documents from the MoWCSC highlight psychosocial and psychological interventions for populations with special needs such as survivors of violence, crime, human trafficking and natural disasters. This includes coverage of fees for psychosocial consultation, psychological evaluations, hospital treatments, psychological treatments and psychotherapies. Psychosocial counselling provision was established for those put in shelter during the COVID-19 pandemic (Government of Nepal, 2020a).

Several documents from the MoHP highlight community-based mental health interventions. These include free basic health services for priority mental health conditions (depression, psychoses, alcohol use disorder [AUD], conversion disorder, and epilepsy), and provision of 11 free psychotropic medications to be prescribed by trained health workers. To implement this, strategies and operations guidelines have provisioned for the World Health Organization’s mental health Gap Action Program adapted training for primary care workers (also termed Module 2 training on mental health) (World Health Organization, 2008). Psychosocial management is highlighted for depression and AUD (Ministry of Health & Population, 2021). Arrangements for specialized psychiatric services have been expanded beyond tertiary psychiatric facilities to include general hospitals and teaching hospitals across the country (Government of Nepal, 2021a).

Indicators in the National Mental Health Strategy and Action Plan and the Multi sectoral Action Plan for Prevention and Control of Non-Communicable Disease (referred to as NCD strategy and action plan henceforth) focused on increased service coverage (e.g., proportion of people using services, number of municipalities implementing community mental health programmes and number of health workers trained) (Government of Nepal, 2021b). However, assessment of quality of services and indicators related to it was missing from the strategies. Table 4 shows interventions that have been highlighted in the policies among key areas and populations.

**Table 4.** Interventions highlighted in policy documents in key areas and populations

Key areas of interventions	Types of interventions and services
Community mental health	Supervision with support from teaching hospitals and provincial hospitals Develop and expand the concept of tele-mental health Promote specialized mental healthcare services through health insurance and tele-mental health services
Integration of mental health in regular public health programmes	Screening of depression and suicide among pregnant and new mothers Drug addiction and mental health to be included in the adolescent sexual and reproductive health programme Assessment of mental health status and counselling services for patients with leprosy, HIV and AIDS Provision of protection or rehabilitation homes for the protection of the reproductive health of women who are mentally disabled
Expansion of mental healthcare to targeted/vulnerable/at risk groups	Revise the community mental healthcare package 2017 to include adolescent mental healthcare in the community and include early detection, promotion and prevention of intellectual and psychosocial disabilities through screening, orientations and rehabilitation Early detection of mental health issues among senior citizens, increased access to social security Screen citizens in foreign employment for mental health issues and provide treatment through insurance and tele-mental health Prevention, promotion programmes at schools against alcohol and drug use, treatment and rehabilitation in collaboration with security mechanisms Suicide: prevention of suicide and promotion of mental health- develop suicide prevention programmes, pesticide control in collaboration with ‘concerned sectors’, media awareness in reporting suicide cases, suicide registry Prisoners- screening of prisoners for mental health condition and treatment Survivors of sexual violence/trafficking/trauma- ‘To make one-stop crisis management center operation and multilateral partnership effective’ and provisions for psychosocial services and therapies Survivors of emergencies – provide psychological first aid and treatment for affected populations Juvenile justice – child taken under control for offence should be referred to psychologists for evaluation and counselling services
Human resources development	Train primary healthcare workers in the detection, treatment and referral of priority mental health conditions Provision of scholarships for study of specialized psychiatry Development and implementation of training packages for school nurses to promote inclusion of mental health in school health programmes Training of teachers on mental health and psychosocial issues Review of curriculum of health education

#### Right to exercise legal capacity and right to personal liberty and security

The sub-theme includes safeguarding PWLEs against detention and treatment without consent, and support to exercise their legal capacity. Here legal capacity is defined as the capacity to have the rights, to understand and exercise the rights (Bantekas *et al.*, 2018).

The Impeachment Act defines ‘incompetence’ as the inability to discharge his/her duties due to physical or mental reasons and so not having legal capacity (Government of Nepal, 2002a). Incompetence is determined by a medical board consisting of three concerning specialists who carry out a medical examination. The National Civil Code Act describes a person with competency as any person who attains 18 years except in cases (amongst others) of those who are ‘*of unsoundness of mind*’, i.e., being incapable due to physical and mental ill health of knowing the act done by oneself and its consequences (Government of Nepal, 2017d). Incompetent persons do not bear any legal obligation, and the exercising of their right can be done only with the consent of the guardian/curator or through the guardian/curator. The act mentions that determination of competency is done by the court without providing any detail on how the determination is made. This discrepancy or lack of clarity in determining incompetence within policies makes the implementation of such policies open to situational and contextual factors thus subjecting it to more discrimination.

The National Civil Code Act states that no discrimination shall be made in the application of general law on the grounds of (amongst others) physical condition but does not mention mental health conditions (Government of Nepal, 2017d). The Act mentions that consent given by a person of ‘*unsound mind*’ due to mental illness if unable to understand the characteristic fault and consequence of the consent will not be considered as consent. This provision on consent is reflected in numerous policy documents: the Public Debt Act (Government of Nepal, 2002b) mentions that if the owners of the bond are ‘insane’, their mother/father/husband/wife may be assigned the protector. Payment of principal and interest of a bond of an ‘insane’ person is done to the guardian and the payment can be made to the person themselves if they submit a certificate from a medical board mentioning details of their wellness. The Public Health Service Act too mentions the provision of health services without informed consent if the service recipient is not in a condition to give consent (Government of Nepal, 2018f). No form of assisted decision-making was mentioned in any of the documents.

#### Freedom from torture or cruel, inhuman or degrading treatment or punishment, and from exploitation, violence, discrimination and abuse

This sub-theme includes provisions in policy documents relating to the right to be free from all forms of abuse and neglect, free from forced treatments and surgeries or Electro Convulsive Therapy (ECT), not subjected to experimentations without consent, and safeguards to prevent torture, cruel treatments and abuse.

The Constitution of Nepal provisions citizens with disabilities to have the right to live with dignity and honour and have equal access to public services and facilities (Government of Nepal, 2015). The National Penal Code Act mentions if the offence was committed against (among others) ‘… a person being of unsound mind by reason of physical or mental illness or a person incapable of defending himself or herself because of disability …’ then it is aggravating the gravity of offence (Government of Nepal, 2017e). The gravity of offence is reduced if the offender has diminished capacity because of physical or mental disability. In the Army Act, accused personnel unable to defend themselves due to ‘*mental insanity*’ during the trial by the court martial may be held in a mental hospital or in custody or in any appropriate safe place (Government of Nepal, 2006). They can be released following recommendation of the chief of the mental hospital or prison ‘…stating that the person might not be harmful to him/herself or anyone else after release’.

The Police Act states that one of the duties of police employees is to ‘… take charge of lunatics and persons who are dangerously intoxicated and cannot look after themselves’ (Government of Nepal, 1955). Similarly, the Prisons Act has provision for arrangements to keep detainees or prisoners who are ‘*insane and half minded*’ separated and kept in different parts as far as possible (Government of Nepal, 1963b). These provisions are in contrast with the Disability Act that states that any person with psychosocial and mental disability shall not be detained in the name of treatment or protection (Government of Nepal, 2017a). There was a notable absence of any policies that discussed safeguards for forced treatments and abuses at the health facilities for persons with mental health conditions.

Several policies had provisions safeguarding persons with mental health conditions from discrimination. The Public Health Service Act prevents discrimination in treatment because of disability (Government of Nepal, 2018f), and the Crime Victim Protection Act (Government of Nepal, 2018a) prevents discrimination in the justice process on the grounds of physical and mental disability. However, the Right to Employment Act protects against discrimination based on caste or ethnicity, but no health conditions or disability are mentioned (Government of Nepal, 2018g). Some legal documents disqualified or restricted persons with mental health conditions from positions or services (see Table 5).

**Table 5.** Examples of policies that disqualify or restrict individuals with mental health conditions from positions or services

**Citizen Investment Trust Act, 2047 (1991) and Cooperatives Act (2017**): Person not eligible for nomination or election for positions within organization/institution if ‘insane or mad’, ‘mentally incapable’. **Nepal Citizenship Act 2063 (2006**): Ineligible to acquire citizenship by naturalization if not mentally fit and healthy. **Civil Service Act, 2049 (1993**): Early retirement given to personnel who is certified by medical board to be unable to serve regularly due to ‘physical or mental diseases’. **Local Level Election Act (2017**): Ineligible to be candidate if of ‘unsound mind’. **Nepal Airlines Corporation Act (1963**): Disqualification of General Manager and Director if a person is an ‘insane or mentally unbalanced’. **Judicial Council Act, 2073 (2016**): Ineligibility for appointment in the Post of Judge if ‘having mental disorder’. Termination of the post if the medical board determines that the judge is unfit to perform the duty due to physical or mental illness.

#### Right to live independently and be included in the community

This sub-theme captures policies that support accessing financial resources to live in the community; access to education and employment; right to participate in political and public life; right to participate in social, cultural, religious and leisure activities; and fulfilling social and personal lives.

Policies had specific provisions for persons with disabilities (including psychosocial and mental disabilities) relating to participation in sports, rehabilitation and livelihood programmes and arrangement of sports competitions to ensure enjoyment of leisure and livelihood activities (Government of Nepal, 2010a, 1992, 2010b). However, the National Civil Code Act states marriage cannot take place if the person has leprosy, incurable severe diseases, hearing and visual disability, HIV or is of ‘unsound mind’ (Government of Nepal, 2017d). The Local Level Election Act restricts persons of ‘unsound mind’ from participating in elections as a candidate (Government of Nepal, 2017c).

The Disability Act has provisions for people with mental health and psychosocial disabilities with entitlements based on severity or socio-economic conditions (Government of Nepal, 2017a). This includes monthly cash allowance; free healthcare and assistive products; education allowance; concession on fees for public transportation; or tax exemption on income, housing, or vehicles.

### Integration and consistency with national policy and the legislative environment

In the absence of a national mental health act, we reviewed other health and social policies to understand the extent of inclusion of the mental health agenda and provisions that are applicable to persons with mental health conditions.

All citizens have the right to free basic health services from the State according to the Constitution (Government of Nepal, 2015). The Public Health Service Act has focused on the inclusion of mental health into basic health services (Government of Nepal, 2018f). The Standard Treatment Protocol for Basic Health Services Package includes pharmacological and psychosocial interventions to be provided by primary health facilities (Ministry of Health & Population, 2021). The National Health Policy addresses mental health as a condition of concern and ensures access to mental health and psychosocial services from local health facilities by promoting knowledge and skills transfer in the primary hospitals (Government of Nepal, 2019a).

During the review period, two of the seven provinces (Gandaki and Karnali) passed their own provincial health policies. The Gandaki Province Health Policy has mentioned outpatient and inpatient services for mental illness in district hospitals; programmes to control drugs and alcohol use; prevention, promotion, diagnosis and treatment of mental illnesses in urban health clinics; and the expansion of specialist services for mental illness (Government of Nepal, 2021e). However, the policy does not specify a community mental healthcare package or the provision of mental health services below the district level. The Karnali Province Health Policy focuses on the expansion of mental health services at all levels with specialist health camps to be conducted periodically in rural areas. It addresses the establishment and management of rehabilitation centres for short-term management of (among others) PWLEs who are helpless (Government of Nepal, 2019d). It acknowledges the role of the provincial government to provide quality and cost-effective services to PWLEs.

The National Health Insurance Act has included the coverage for selective mental health conditions (conversion disorder, severe depression, bipolar disorder, epilepsy and schizophrenia) (Government of Nepal, 2018c). However, the benefit packages only cover medical management covering the cost of the psychotropic medicines for these conditions. Mental health indicators are included in the multi-sectoral action plan for NCDs such as a 10% reduction in harmful alcohol use, and an increase in service coverage by 25% (specifically for psychosis and depression) (Government of Nepal, 2021b). Similarly, policies of other health conditions such as leprosy, HIV and AIDS have included provisions for psychosocial counselling and psychiatric services (Government of Nepal, 2018d, 2011, 2014). This shows that mental health is being integrated to some extent into these broader health policies.

Employment and housing policies do not address mental health conditions. Other policies related to children, domestic violence, survivors of human trafficking and social security have provisions specifically for persons with disability. They have added provisions for counselling or therapies for their respective beneficiaries. The Disability Act has secured fundamental rights of persons with disabilities with an additional clause specific to mental health conditions that include treatment at the local community of their choice, rehabilitation and treatment of persons with psychosocial disabilities who have been abandoned by their families, and not to keep such persons in prison (Government of Nepal, 2017a).

Inclusion of mental health in the development agenda and plans were present but scarce. The National Planning Commission (NPC) which produces the 5-year national plan in its 15th Plan and the National Health Sector Strategy implementation plan have identified mental health as a major problem in health and nutrition, and have mentioned plans for expansion of access to mental health services at all levels and systematic development of alternative medicines including psychological counselling along with yoga, meditation and Ayurveda (Government of Nepal, 2020b, 2016).

### Coordination and inter-sectoral collaborations

A dedicated governance mechanism to look into mental health at the federal, provincial and municipal levels has been proposed in the national mental health strategy and action plan (Government of Nepal, 2016). However, this is not reflected in the provincial-level health policies that were included in this review. The National Mental Health Strategy and Action Plan 2020 identifies federal, provincial or local government as responsible and coordinating bodies for all the major activities and has highlighted multilateral and multisectoral coordination and collaboration with the government, non-governmental and private sectors for mental health services (Government of Nepal, 2021a). However, specific action plans for these collaborations are missing. There is a specific strategy for the formation of mental health self-help groups for necessary discussion and decision-making on health treatment and rehabilitation needs. However, there is no mention of any specific action plan on who should form these groups, how they will be formed or collaboration with advocacy groups and service user or caregiver organizations for the implementation of the major activities.

Alternatively, the NCD action plan and strategy have included some strategies for mental health and have delineated clear action plans, target dates and agencies responsible (Government of Nepal, 2021b). For example, for the reduction of harmful use of alcohol, the first action plan is the revision of the alcohol control policy and a bill that is targeted to be completed by 2022 by the MoHP in partnership with the Ministry of Home Affairs (MoHA) and the Ministry of Law, Justice and Parliamentary Affairs.

### Financing

The policy documents included in the review did not clearly specify the sources and mechanisms of funding required to finance the implementation of mental health programmes. The Public Health Service Act has mentioned that the financial burden of basic health services (that includes basic mental health) lies on the government (Government of Nepal, 2018f). However, the National Mental Health Strategy and Action Plan has specified action plans that go beyond basic health services. Although the National Health Insurance Act has provisioned additional medications and clinical services not covered by the basic health services, the action plans and interventions do not mention evidence-based financing or cost-effectiveness analysis of prevention, promotion and psychosocial services (Government of Nepal, 2018c).

The operational guidelines for province and local health programmes have detailed activities and budget distribution by the government for various health programmes annually (Government of Nepal, 2019c, 2021c). The guidelines included in the review show ad hoc allocation of a budget with large variations from one year to another for mental health–related programmes. Since 2018, programmes in mental health and its financing were included mainly under the Epidemiology and Disease Control Division, and the activities focused on training of health workers under the community mental healthcare package. Funding for the training of health workers was allocated every year since 2018, although the amount of budget varied. Another programme that received funding for multiple years was the mental health programme for prisoners. Awareness programmes for school health nurses on adolescent mental health and awareness-raising programmes started to be funded in 2018–2019; however, those were not continued in the following year. However, mental health training for school nurses was planned and budgeted for 2021–2022 but not followed up in 2022–2023. A similar case was seen for the purchase of psychotropic medications in each province – which was budgeted only in 2021–2022 but not in the previous or following years.

## Discussion

We reviewed 89 public policies, strategies, legislations and guidelines endorsed by the Government of Nepal to assess and understand how structural stigma and discrimination are manifested in these documents. The review identified few positive changes in policies. First, the inclusion of the mental health agenda has been increasing over the years – especially in post-2015 policies. This momentum can be attributed to the heightened focus on psychosocial and mental health field in the aftermath of earthquake, aligning with the ‘building back better’ concept (Chase *et al.*, 2018). Furthermore, mental health and psychosocial wellbeing are not only included in health policies but are increasingly addressed in social welfare policies, such as those pertaining to children, senior citizens, gender-based violence or trafficking.

Second, compared to the archaic policies (not included in the review), there were notable changes in the language and words used in the recent policies. Words such as *baulaahaa/paagal* (mad/insane) were commonly used to indicate persons with mental health conditions in major policies such as previous *Muluki Ain, 2020* (civil code, 1963) with discriminatory provisions such as chaining and imprisonment of people with mental health conditions until they become ‘normal’ after treatment (Government of Nepal, 1963a). Such provisions no longer exist in current policies, and in many policies, stigmatizing terms such as lunatic or insane have been replaced by legal terminology such as ‘of unsound mind’.

Third, increased attention is given to integrating mental health into primary healthcare and basic health services. This aligns with WHO recommendations for increased coverage and a reduced treatment gap in mental health (World Health Organization, 2008). The NPC periodic plans demonstrate evolving priorities for mental health. The 12th, 13th and 14th NPC plans (2011–2019) focused on specialist services for people affected by conflict, disasters and violence; community outreach through mental health camps; and inclusion of mental health in health awareness programmes. However, the 15th plan (2019–2024) has delineated a clear working policy to expand mental health services at all levels through community health systems complementing the National Mental Health Strategy and Action Plan target to expand community mental health programmes in 500 municipalities by 2025 (Government of Nepal, 2020b, 2021a).

Despite these positive changes, the review identified several important and continuing gaps pertaining to structural stigma and discrimination, which limits the rights of persons with mental health conditions. These notable gaps and recommendations to address these gaps are described as follows:

### Use of stigmatizing language and lack of clear definitions

Terminology and language in policies are indicators of structural stigma, as they expose the legal environment and confirm an institution’s culture and attitudes (Corrigan and Rao, 2012). While some progress has been made in revising stigmatizing language, labelling terms such as *baulaaeko* (insane), imprecise language (mental disease, drug abuse), devaluing terms (mentally incapable/handicapped, mentally incapacitated) and dated terminology (mental asylum) persist in several policies. Similarly, some amended policies have replaced the previous stigmatizing words ‘mentally unstable/insane’ to ‘of unsound mind’. Although this terminology and its definition encompasses both mental and physical incapacity, the policy and strategies formed on its basis primarily affects those with mental health conditions, reinforcing negative attitudes and behaviours, resulting in barriers to healthcare and legal services due to self, public and structural stigma (Szwed, 2020).

Another notable gap was the lack of definitions for mental health–related terminology. Due to the lack of mental health–specific legislations or policies, definitions on mental health, mental disability, psychosocial disability, mental disorders/conditions or mental health services were absent, although many documents used these terms and have specific provisions related to them. For instance, the Social Security Act includes disability benefits for persons with disabilities, where one of the conditions listed is ‘mental unsoundness’, but it remains unclear which mental health condition or functioning level qualifies for the benefit (Government of Nepal, 2018i). The Disability Act of Nepal incorporates mental and psychosocial disability and defines disability as a ‘long-term impairment’ (Government of Nepal, 2017a). Yet, questions persist about whether the act captures people with non-chronic mental health conditions that can still be debilitating and discriminatory. The definition for mental or psychosocial disability in the policy pertains to ‘*problems arising in brain*’ that hinder intellectual activities. Although there may be room to argue that use of biomedical definitions and classifications of mental health conditions may increase stigma, lack of clear definitions can make policies and strategies less actionable and measurable. Similarly, the use stigmatizing or vague terminology may have contributed to the use of inconsistent language regarding individuals with mental health conditions in different policy documents.

*Recommendations*: Immediate policy revisions are essential to eliminate stigmatizing language and promote person-centred language. A regular reviewing process should be established to prevent retention of problematic terms. There is an urgent need to add standard definitions of key terminology in mental health–related policy documents to ensure consistency and understanding. Including PWLEs in policy revision and development can enhance their rights and reduce structural stigma in policies.

### Inconsistencies within and between policies limiting the rights of people with mental health conditions

Several inconsistencies were found both within and between the policies. The Disability Act states that a person with psychosocial or mental disability not to be held in prison for treatment or protection. However, the Army Act allows holding individuals in prison or mental hospitals until the recommendation of the mental hospital or prison chief for release (Government of Nepal, 2006). Similarly, the Police Act permits taking charge of ‘lunatics’ unable to care for themselves as part of their ‘duties’ (Government of Nepal, 1955). Some policies disqualify a person from a position or candidacy if ‘insane’ or ‘of unsound mind’ (Government of Nepal, 2017c). Additionally, the National Civil Code Act prohibits persons with mental health conditions from marrying (Government of Nepal, 2017d). These provisions contradict health and social policies like the Disability Act and the Constitution of Nepal, which grants equal access to public services and facilities for persons with disabilities. Contradictions are observed between national and provincial health policies. While the national strategies and action plans for mental health and NCDs focus on expanding community-based care, Gandaki Province emphasizes out- and in-patient services in district hospitals and urban clinics only, and Karnali Province prioritizes specialist health camps in remote areas (Government of Nepal, 2021e, 2019d).

Another inconsistency noticed was lack of provisions for people with substance use disorders in many health and mental health policies. Most of the health policies focused on AUDs specifically, while sparingly discussed any provisions for promotion of health and wellbeing of people with drug/substance use. This could be because use and possession of drugs is criminalized under Nepal’s law and falls under the directive of MoHA rather than Ministry of Health or Social Welfare. This substantially limits the rights of people living with substance use disorder, restricting their access to health and rehabilitation.

*Recommendations*: Reviewing policies for inconsistencies within and between national policies is crucial. A mental health–specific act or policy could establish a standard for formulating mental health provisions in other policy frameworks. In the absence of such policies, closely related ones like the Disability Act that are based on international conventions such as the UN Convention on the Rights of People with Disabilities (UNCRPD) can serve as a standard to promote the rights of individuals with mental health conditions.

### Inadequate integration of a mental health agenda in the broader national policy framework

Inclusion of low-priority programmes like mental health into broader health or development policy facilitates optimizes available resources and enhances successful implementation of mental health programmes (Faydi *et al.*, 2011). Despite the mental health strategy’s call for integrating mental health agenda into relevant policies, the current policy framework exhibits significant gaps. While some integrations have occurred, such as in the basic health service package, standard treatment protocol and the NCD action plan, development policies related to social security, employment, education, housing, poverty, and sexual and reproductive health remain noticeably silent.

*Recommendations*: Highlighting the intersectionality of mental health in various health and social sectors and thereby facilitating the achievements of national and international goals such as Sustainable Development Goals may facilitate the integration of mental health into multiple development policy frameworks.

### Deviations from international protocols and conventions

Several deviations from the guiding principles of UNCRPD and Universal Declaration of Human Rights were seen in the policies. Instances were found where legal capacity was undermined based on ‘incompetence’ in the policy documents like Impeachment Act (Government of Nepal, 2002a) and National Civil Code Act (Government of Nepal, 2017d). In the absence of a mental health act, policies lack provisions against involuntary and forced treatments that are common in patients with mental health conditions, where consent cannot be provided (Government of Nepal, 2018f). However, no assisted decision-making safeguarding the rights of persons with disabilities are mentioned in the policy documents, in contrast to the Article 12 of the UNCRPD (Alston, 2017).

*Recommendations*: As a country that has ratified UNCRPD and its optional protocol, Nepal must adhere to and report on the compliance of state policies with the protocol. UNCRPD in the past has raised issues regarding assisted decision-making in policies, and Nepal committed to establish legal measures for supported decision-making to ensure fundamental rights (United Nations Committee on the Rights of Persons with Disabilities, 2018). Central and provincial mental health units and legal bodies should prioritize policy review in line with UNCRPD and fulfil the commitments to the UN. Involvement of advocacy groups in policy reviews and evaluation can enhance compliance with the and meet the needs of PWLEs. Similarly, international agencies, UN bodies, advocacy groups and national stakeholders can facilitate in overseeing effective policy revision and implementation.

### Lack of reliable financing mechanisms for the implementation of mental health strategies and action plans

Feasible financing mechanisms are imperative for effective policy implementation. However, the reviewed policies lack cost-effective interventions, sustainable budget planning and identification of funding sources. Although holistic services and training in effective psychosocial support and counselling are prioritized, the annual health programme operation guidelines allocate limited resources for psychosocial support and rehabilitation, prioritizing prescriber training and psychotropic medication purchases (Government of Nepal, 2019c, 2019b, 2021c, 2021d). Furthermore, while disease burden assessment is reported in the policies, there remains a gap in assessing financing arrangements and micro-fiscal aspects that are critical for successful policy implementation. Consequently, budget allocation for mental health programmes appears ad hoc and fluctuates annually, lacking sustainability, as evident in the annual operation guides.

*Recommendations*: To ensure successful implementation and sustainability of the mental health action plan, feasible funding mechanisms need to be identified. Inter-sectoral collaboration and budget planning among responsible sectors for mental health policy implementation can facilitate cost sharing, preventing undue burden on one sector. The inclusion of mental health into ongoing health insurance schemes has been identified as an effective strategy for low- and middle-income countries (Chisholm *et al.*, 2019). Although in a nascent stage, Nepal has currently scaled up its national health insurance scheme that includes limited specialist mental health services (Government of Nepal, 2018c). Exploring insurance scheme reforms to include evidence-based treatments, securing alternative domestic resources (e.g., excise taxes) and leveraging support from international communities can offer additional funding avenues for mental health programmes. Designing pilot projects that evaluate the feasibility, cost-effectiveness and scaling-up of the interventions should be prioritized by the government and related stakeholders.

## Limitations of the study

The review included unclassified policy documents from three government websites. Although we selected the three main government bodies responsible for health, social welfare and legal codes, other websites and sources may have relevant documents, leading to potential gaps in the review’s comprehensiveness. For instance, National Adolescent Development and Health Strategy, 2018, was not identified during the review process (Government of Nepal, 2018b). Similarly, we missed a large body of legal documents pertaining to Drugs and substance use, which are listed under MoHA rather than Ministry of Health. Additionally, we were unable to categorize which policies supersede others when there are conflicting rights and restrictions between policies. We tried to address this by reaching out to relevant stakeholders and policymakers to share relevant policy documents and check for the inclusion of overridden documents. We also acknowledge subjectivity of the review process, despite involving multiple reviewers in the process and using framework guided by tools like WHO checklist for policy analysis and QualityRights. We tried to address this through specific inclusion criteria, search strategies and periodic review of subsets of controversial policies. Decisions were made through discussion and consensus. The analysis did not cover policy implementation, leaving room for future research on implementation status.

## Conclusion

This review assessed public policies, legislations and guidelines endorsed by the Government of Nepal to understand how structural stigma and discrimination. Positive attention to mental health in recent social welfare and general health policies amended terminologies and attention to integration of mental health into basic health services. However, several gaps that contracted the rights of PWLEs were observed, including retention of stigmatizing terminology, unclear definitions, inconsistencies within and between policies that contracted rights and liberties of PWLEs, and deviations from international conventions such as. Moreover, the mental health agenda was missing in larger development policies, and financing mechanisms were lacking for sustainable implementation. Routine policy revisions to amend stigmatizing language and provisions that restrict the rights of PWLEs, integrating the mental health into larger development policies, and aligning with international protocols may help reduce mental health–related structural stigma and discrimination in Nepal.

## Data Availability

Additional data and materials related to the review is available from the corresponding author dristy.1.gurung@kcl.ac.uk
