# Mental health–related structural stigma and discrimination in health and social policies in Nepal: A scoping review and synthesis – ERRATUM

**DOI:** 10.1017/S2045796023000847

**Published:** 2024-01-04

**Authors:** D. Gurung, M. Neupane, K. Bhattarai, B. Acharya, N. C. Gautam, K. Gautam, S. Koirala, K. Marahatta, P. Gurung, K. B. Khadka, B. A. Kohrt, G. Thornicroft, P. C. Gronholm

DOI: https://doi.org/10.1017/S2045796023000823, Published by Cambridge University Press, 13 December 2023

The original published article contained incorrect affiliations. This has been corrected and the article republished.

The publisher apologies for the error.

## References

[ref1] Gurung D, Neupane M, Bhattarai K, et al. Mental health–related structural stigma and discrimination in health and social policies in Nepal: A scoping review and synthesis. *Epidemiology and Psychiatric Sciences*. 2023;32:e70. First published online 13 December 2023 doi:10.1017/S2045796023000823 PMC10803190 38086740

